# Cognitive Variables in Social Anxiety Disorder in Children and Adolescents: A Network Analysis

**DOI:** 10.1007/s10578-021-01273-9

**Published:** 2021-10-27

**Authors:** Felix Vogel, Julian Reichert, Daniela Hartmann, Christina Schwenck

**Affiliations:** 1 Department of Special Needs Educational and Clinical Child and Adolescent Psychology, Justus-Liebig-University of Giessen, Otto-Behaghel-Straße 10 E, 35394 Gießen, Germany; 2 Medical School Hamburg, University of Applied Science and Medical University, Hamburg, Germany; 3 Department of General Internal Medicine and Psychosomatics, University Hospital Heidelberg, Heidelberg, Germany

**Keywords:** Social anxiety disorder, Network analysis, Dysfunctional cognitions, Negative expectations

## Abstract

Clark and Wells’ prominent model of social anxiety disorder (SAD) assumes that cognitive variables such as negative expectations or dysfunctional cognitions play a central role in the symptomatology of SAD. In contrast to adults, it is less clear how well the cognitive model can be applied to children and adolescents. A network analysis with seven nodes was conducted to explore the importance of cognitive variables and their interaction with symptoms of SAD based on N = 205 children and adolescents (8–18 years, *M* = 11.54 years). Cognitive variables had a high but differential impact within the positively connected network of SAD. Dysfunctional cognitions were most strongly connected within the network. Dysfunctional cognitions, as predicted by Clark and Wells’ model, seem to act as a hub affecting several symptoms. The association between negative expectations and avoidance indicates that negative expectations may particularly contribute to the maintenance of SAD.

**Supplementary Information:**

The online version contains supplementary material available at 10.1007/s10578-021-01273-9.

## Introduction

Social anxiety disorder (SAD) is characterized by an intense and persistent fear of social situations and fear of being evaluated by others [1]. SAD is one of the most common mental disorders of childhood and adolescence, with life-time prevalence rates ranging from 7 to 9% [2, 3]. Affected individuals show impairments in academic and social functioning [4, 5] and are at a higher risk of developing comorbidities [3, 5]. Notably, the most common onset of SAD is during childhood and adolescence, with a high risk of being persistent into adulthood [6–8]. This emphasizes the importance of identifying factors that contribute to the maintenance of the disorder and thus represent promising targets for treatment early on. The DSM-5 [1, 9] conceptualizes SAD as a set of symptoms including fear of social interactions, fear of being observed, fear of performing in front of others, and avoidance of social situations. Attending social situations is associated with the occurrence and endurance of the fears mentioned here. In addition, fear of acting in an embarrassing way or fear of showing bodily anxiety symptoms (physiological symptoms) such as blushing, trembling or sweating, are part of SAD [1]. In a factor analysis of social fear in children and adolescents, it was shown that fears are best represented by three domains, namely interaction, observation, and performance [10]. Research further indicates that all three domains of fear are associated with avoidance behavior and thus are likely to contribute to the maintenance of SAD [10, 11].

### Cognitions in Children and Adolescents with SAD

Beyond the symptoms described in the DSM-5 [1], models of SAD suggest additional variables immanent to SAD [12–15]. One of the most prominent models of SAD, is the model by Clark and Wells (1995) [12]. The model assumes that individuals with SAD hold negative expectations regarding social situations. When attending a social situation, these negative expectations are activated and lead to dysfunctional cognitions during the social situation, which, in turn, activate physiological and other cognitive symptoms. As an example, imagine a child who has the negative expectation (pre-event processing) “When I talk in front of others they will laugh at me,” which then leads to the dysfunctional cognition “If I have to talk in front of others, I must always speak eloquently (so that nobody laughs at me).” During the situation, physiological symptoms (e.g., trembling of voice) and an increased perception of these bodily reactions (self-focused attention) occurs, which, in turn, amplifies the fear that others might laugh. In the aftermath of the social situation, the child ruminates about the past social situation negatively (post-event processing), which in turn strengthens the negative expectations regarding similar situations. Consequently, social situations are more likely to be avoided due to such negative expectations [16]. In this chain of cognitive and physiological processes, dysfunctional cognitions play a central role as they affect several other SAD symptoms. Thus, dysfunctional cognitions are viewed as a hub.

### Research on Cognitions in Children and Adolescents with SAD

While negative expectations and dysfunctional cognitions are already well researched in adults with SAD and are central targets of interventions [14], few and inconsistent findings exist in this regard in children and adolescents [17]. However, the sparse research consistently indicates that socially anxious children are more likely to expect that future social situations will have a negative outcome compared to non-anxious children [17–20]. Research regarding the occurrence of dysfunctional cognitions during a social situation in socially anxious children and adolescents is, surprisingly, much more inconsistent than findings regarding negative expectations [17]: some studies indicate a more frequent occurrence of dysfunctional cognitions in social situations in children with SAD compared to healthy children [18, 19], whereas others do not show any differences [20–22]. Although it is still unclear whether dysfunctional cognitions are more frequent in children with SAD, studies that have looked at dysfunctional cognitions concerning social anxiety using path-analytical or regression-analytical approaches in larger community samples show first evidence for the relevance of dysfunctional cognitions in children and adolescents. These studies demonstrate that dysfunctional cognitions are the second strongest predictor of social anxiety in children and adolescents [23, 24] and, within a set of other variables of cognitive SAD-models, are able to explain almost half of the variance in social anxiety [23, 24]. In line with the cognitive model of Clark and Wells (1995) [12], dysfunctional cognitions are also predicted by pre-event processing, confirming that negative expectations may lead to dysfunctional cognitions during a social situation. Clark and Wells’ (1995) [12] cognitive model further mentions factors such as post-event processing and self-focused attention which are also identified as relevant for children and adolescents in this context [25–28]. Thus, cognitive variables might be contributing factors to the maintenance of SAD in children. Despite the assumed importance of cognitive variables included in models of SAD, there are almost no studies examining the association of cognitive variables and circumscribed symptoms of SAD such as social fears, physiological symptoms, and avoidance behaviors in children and adolescents. One study found a correlation between expectations of one's performance and avoidance behavior in a community sample of adolescents [29]. Another study indicates that experimental induction of negative expectations in children results in higher state anxiety levels during the task [30], suggesting a direct relation between expectations and social fears. Although the influence of cognitive variables on symptoms of SAD in children and adolescents is largely unexplored, the aforementioned cognitive variables of Clark and Wells’ (1995) [12] model are already considered in children and adolescents in the context of cognitive behavioral therapy (CBT) [14, 31], for which research indicates a promising effectiveness in the treatment of SAD [32]. However, we only know little about the effect of targeting individual cognitive variables in CBT [14]. Since first evidence shows that targeting specific cognitive variables may actually increase efficacy [33, 34], it seems important to gain a better understanding of the contribution of individual cognitive variables to SAD in children. To date, no study has examined the cognitive variables described here in in relation to physiological symptoms, social fears, and avoidance behavior. Therefore, (i) it is largely unclear whether cognitive variables are connected to all these above-mentioned symptoms of SAD in children and adolescents. Additionally, (ii) the overall importance of specific cognitive variables in children and adolescents in the context of SAD and (iii) the influence of these cognitive variables on individual symptoms of SAD are largely unknown. Knowledge of the differential influence of cognitive variables on symptoms of SAD, for example whether dysfunctional cognitions actually act as a central hub with global influence on many SAD symptoms, could contribute to the question whether Clark and Wells' (1995) cognitive model can also be applied to children and adolescents. Furthermore, a nuanced understanding about cognitive variables in children and adolescents with SAD could help develop meaningful treatment strategies.

### Network Theory

In order to gain a fine-grained understanding of the importance of cognitive variables and their interaction with symptoms of SAD, the network theory of psychopathology [35–37] represents a promising approach. In the network theory of psychopathology, a network consists of nodes (which represent symptoms or variables) and edges (connections between symptoms or variables). The network theory of psychopathology represents a new way of conceptualizing psychopathology, in which it is assumed that there is no single underlying pathomechanism or common cause of the disorder and all its symptoms [35]. Instead, a mental disorder emerges through influences and interactions between symptoms and related variables that activate each other. Through mutual activation and reinforcement of symptoms and variables among themselves, a stable state of mental disorder arises, so that the network itself *constitutes* the mental illness (for a theoretical overview see [35, 37]). Based on network theory, various aspects can be investigated to provide information about the properties of the analyzed network and thus about the mental disorder itself (for a methodological overview see [38, 39]). Related to the present study, network analysis can contribute as follows: First (i), to gain insight whether above-mentioned cognitive variables and individual symptoms of SAD are connected at all and form a network, one can examine the network structure or architecture, that is, whether the connections between the symptoms of SAD and cognitive variables tend to be strong or weak, positive or negative [40]. Strong and positive connections between the symptoms of SAD and cognitive variables would indicate that the included nodes are meaningful symptoms and cognitive variables of SAD, and that these, in combination, represent a stable state and thus a persisting mental disorder [35]. Second (ii), regarding the question of how strong the influence of individual cognitive variables or symptoms of SAD is within the network overall, one can consider centrality measures and predictability [38, 41]. As the most prominent centrality measure, *strength* would indicate how strongly a cognitive variable or symptom is directly linked to all other symptoms and cognitive variables in the network. It indicates this symptom’s or variable’s relative importance within the network [38]. This would provide a quantification of the importance of the cognitive variables and symptoms of SAD relative to each other, so that the analysis is not solely dependent on visual exploration [38]. Predictability, which is equivalent to the explained variance of the nodes among themselves, represents an absolute measure of the symptoms’ and variables’ importance [41, 42]. High predictability of a certain symptom (e.g. avoidance behavior) would indicate that avoidance behavior can be well predicted by all other symptoms and cognitive variables that are connected to it. According to network theory, nodes with a high predictability are considered to make an important contribution to the maintenance of the disorder [41]. Deactivation of this cognitive variable or symptom (e.g. by treatment) is considered to be associated with destabilizing the network itself and thus probably results in the remission of the disorder [42]. Third (iii), the question of whether and to what extent cognitive variables or symptoms of SAD are specifically associated with individual other symptoms or cognitive variables can be addressed by analyzing individual edges between nodes. Individual edges within a network represent significant connections between two symptoms and indicate that symptoms influence each other [35]. By comparing individual edges, it is possible to examine whether a link between two nodes (e.g., between a cognitive variable and a symptom) is particularly strong or weak. If there is a strong positive association between two nodes, network theory assumes that the activation of one node likely activates all associated nodes [36]. Vice versa, the deactivation of one symptom is accompanied by the deactivation of the other symptom [36]. For example, if the two nodes dysfunctional cognition and avoiding social situation were strongly linked, dysfunctional cognition is likely to activate, avoiding social situations or vice versa. The advantages of network analysis include modelling relevant connections between components (e.g., symptoms and cognitive variables) without having to use sum scores. It also allows the influence of individual components within the network to be quantified using objective estimators.

### Network Analyses on SAD

To date, few studies have explored a network only based on SAD symptoms for adult individuals [43–48]. In this context, Heeren et al. [45] found a quantitatively stronger link between symptoms in a sample of adult patients with SAD compared to a healthy control group, but the networks did not differ regarding their structure and centrality. This is consistent with the idea that a certain level of SAD-related symptoms is normative, that each individual can be located on a continuum of severity of social anxiety, avoidance behaviors, or physiological symptoms, and that SAD is present if a certain level of symptomatology is exceeded [49]. Thus, in complement to the categorical classification of the DSM-5, a dimensional approach with healthy or mixed samples can also be applied to analyze symptoms of SAD [49–51]. So far, publications on networks for SAD in adults have neglected cognitive factors or physiological symptoms but focused on anxiety-inducing social situations [43, 45–48] and attentional mechanisms [44].

To date, only one study has reported an SAD network relying on data from adolescents of a community sample [52]. In contrast to networks in adult samples, the authors included different components such as cognitive variables, nervousness in social situations, and avoidance behavior and analyzed the network’s stability over time. As cognitive variables, they included different negative thoughts (e.g., “I worry about doing something stupid or embarrassing”), which formed a symptom cluster (also called community) and were only indirectly related to avoidance behavior through emotional variables (e.g., “I get nervous if I have to perform in public”), which are conceptually close to social fears. At all three measurement points, a negative thought was the component with the highest strength within the network. However, the authors neither differentiated between negative expectations and dysfunctional cognitions nor considered physiological symptoms or different types of social fears. Overall, the results of this study emphasize the importance of cognitive variables for SAD in children and adolescents but do not allow to draw detailed conclusions for the interaction of different cognitive variables as well as their interaction with other important symptoms of SAD.

## Current Study

In the present study, we conduct an SAD network analysis for children and adolescents. We consider a broad spectrum of SAD symptoms, including main fear domains of social anxiety (observational, interactional, performance), avoidance behavior, and physiological symptoms, as well as SAD-immanent cognitive variables. Since a network analysis requires a balance between the number of included nodes and sample size [39], and our analysis is based on an existing set of data, we focus on the three key fear domains [10], the cognitive variables negative expectation and dysfunctional cognition, physiological symptoms, and avoidance behavior. With our network analysis, we want to examine if cognitive variables that are empirically proven to be part of SAD models in adults and were only non-specifically analyzed within a symptom network in adolescents are a hallmark of social anxiety in children and adolescents as well. Because cognitive variables are not commonly considered symptoms of SAD, but in the present study symptoms and cognitive variables will be considered together in a network, both are jointly referred to as *components* in line with Miers et al. (2020). Due to the lack of network studies regarding SAD in children and adolescents and the exploratory nature of the network approach, we do not form specific hypotheses. Instead, we assume (1) that all included components (symptoms and cognitive variables) are positively connected and thus build a symptom network of SAD, as has previously been the case for adults and adolescents [45, 52] and (2) that, based on the initial evidence of the importance of cognitive variables [17, 23, 24, 29, 52], they display a central role within the network, and that dysfunctional cognitions, in particular, are highly interconnected due to their assumed role as a hub. Since Miers et al. (2020) [52] did not differentiate between different cognitive variables, we (3) aim to explore if different cognitive variables (negative expectation and dysfunctional cognitions) are differentially connected to symptoms of SAD.

## Methods

### Sample

We conducted our network analysis based on two data sets from previous studies. For more information on the recruitment and procedure of the first study, one may refer to descriptions in other publications [53][53]. Both children and parents who participated in these studies were informed about the content of the respective study in advance and gave their informed consent prior to their participation. In the first study, *n* = 109 children and adolescents aged 8–18 years completed the Social Phobia and Anxiety Inventory for Children (SPAI-C) [55] as part of a nationwide online study in Germany focusing on anxiety-related cognitions [54] and anxiety levels in social situations [53]. In this study, families were recruited through therapeutic outpatient clinics, internet forums, and newspaper articles. Parents had the opportunity to participate in a draw for 10 vouchers worth €25 each. The study took place from January 2016 to December 2017. In the second study, which has not been published so far, children aged 8 to 12 years completed the SPAI-C as part of a study on social stress and gaze behavior in social situations. The sample was recruited through newspaper articles, schools, therapeutic outpatient clinics, and mailings in the state of Hesse (Germany). Families were compensated with 20€ for their participation. The study took place from January 2018 to March 2020. In this study, n = 95 children completed the SPAI-C. Thus, the current analysis includes a complete data set of a mixed study sample of *N* = 204 (67% female) children and adolescents between 8 and 18 years of age (*M* = 11.54, *SD* = 3.15) with a mean sum score of all SPAI-C items of *M* = 20.00 (clinical cut-off = 20), *SD* = 13.53. In addition to trait social anxiety assessed with the SPAI-C, symptomatology of selective mutism (SM) was assessed in the two aforementioned studies using the Frankfurt Scale for Selective Mutism (FSSM) [56]. Therefore, trait social anxiety and SM symptomatology will be reported for the present sample, although the present network analysis is based only on the items of the SPAI-C. An overview of our sample composition is provided in Table 1. The subdivision into children with high social anxiety (HSA) and low social anxiety (LSA) provides a better overview of individuals with clinically relevant scores on the social anxiety continuum. Subsequent network analyses were conducted for the complete sample. It can be seen that our sample includes a substantial number of individuals with clinically relevant levels of social anxiety (Table 1). Additionally, some of these individuals also exceed the cut-off for SM, which is in line with studies indicating that most children with SM also have a comorbid SAD [57], report elevated trait social anxiety [58] and do not differ on fear-related cognitions compared to children with SAD [54]. Thus, we were able to cover a broad spectrum of the dimension of social anxiety.

**Table 1:** Sample characteristics divided for children with high and low social anxiety

	HSA^a^	LSA^b^	*p*	Comparison
N	94	110		
Age	12.20 (3.67)	10.96 (2.52)	.006	HSA > LSA
Gender (f/m)	70/24	67/43	.040	
SPAI-C-Score	32.58 (8.02)	9.30 (5.65)	> .001	HSA > LSA
Number of children who exceeded the cut-off for SM^c^	65	23	> .001	HSA > LSA

^a^HSA = high-socially anxious: children who exceeded the clinical cut-off on SPAI-C (Social Phobia and Anxiety Inventory for Children); for indication of HSA we used the Cut-off of 20 from SPAI-C;; the the division into these groups serves a better overview of important variables of our sample and we didn’t compare these two groups within our network analysis

^b^LSA = low socially-anxious: children who did not exceed the clinical cut-off on SPAI-C

^c^SM = selective mutism; for indication of SM we used Frankfurter Scale for Selective Mutism with a Cut-off of 6 or 7 depending on development-adapted version

### Measures

Social anxiety symptoms (*performance fear, interactional fear, observational fear, physiological symptoms, and avoidance behavior*) and cognitive variables (*negative expectations and dysfunctional cognitions*) were assessed using the German version of the SPAI-C [55]. The SPAI-C consists of 26 items rated on a 3 point Likert scale ((0) “never or rarely,” (1) “sometimes,” (2) “most of the time or always”) concerning symptoms of SAD as well as related fears and cognitions in children and adolescents. The SPAI-C has a sum score range of 0–52 with a clinical cut-off at 20 [55]. Although the SPAI-C is designed for children and adolescents between 8 and 16; 11 years, the questionnaire can also be used up to age 18, as long as the adolescents are still students [55]. Both reliability (Cronbach’s Alpha = 0.92) and validity of the SPAI-C are considered as high [27, 59]. In the literature, there are different findings regarding the dimensions of the SPAI-C. Here, studies indicate a 1-factor [55], 3-factor [60], 5-factor [61], or a hierarchical 5-factor solution with social anxiety as the upper factor [62]. All factor solutions were found in community samples [55, 62] or mixed samples of children with and without high social anxiety [60, 61].

### Data Analysis

The data sets were merged using IBM SPSS 26.0, and all further analyses were performed with RStudio (RStudio Team, 2020) using the lavaan [63], networktools [64], qgraph [65], and bootnet [66] packages.

#### Item Selection

A central problem of network analysis of psychopathology is that either all items of a questionnaire or a selection of items only based on the items’ content is included in the network analysis. While the former approach can lead to a strong overlap between included components [36], in the latter approach it is often not clear how the selection of items was achieved. Therefore, to prevent too much overlap between components and to base the item selection not only on theoretical but also on empirical criteria, we adopted an approach in which we empirically tested for the components and corresponding items of the SPAI-C we aimed to include in the current network. For this purpose, we conducted a confirmatory factor analysis (CFA) in which we assigned items of the SPAI-C to the seven components (symptoms and cognitive variables described in theory section—see also Table A in Supplements). In this context, the CFA functioned as an empirical tool. The various CFA fit indices indicate whether the seven components included in the network are empirically justifiable compared to factor solutions reported in the literature and the factor loadings show, which item is most representative of each component. For each of the seven components, we chose the item with the highest loading as representative to further reduce redundancy (see Table 2). Items that could not be assigned to a particular component (item 1, 10, 11, 12, 13, 18, 22, and 23) were excluded from the analysis. We then compared the fit indices of the CFA to test whether these seven resulting components prevailed over the factor solutions described in research (1-, 3-, 5-, and hierarchical 5-factor solution). For this purpose, various fit indices [67] were used as measures of comparison (CFI, RMSEA, SRMR). Due to the ordinal scaling of the SPAI-C, we used the maximization likelihood method, which is robust to possible violations of the multivariate normal distribution. Accordingly, to calculate the factors already described in the literature, we used those items that were assigned to each of the described factors in previous studies [55, 60–62]. According to all fit indices, our solution based on the seven components (χ^2^ (116) = 222.722, p < 0.001; CFI = 0.965, RMSEA = 0.067, SRMR = 0.035) showed a descriptively better fit than the other factor solutions (5H factor solution (χ^2^ (203) = 452.915, p < 0.001, CFI = 0.916, RMSEA = 0.078, SRMR = 0.048); 5-factor solution (χ^2^ (198) = 434.193, p < 0.001, CFI = 921, RMSEA = 0.076, SRMR = 0.045); 3-factor solution (χ^2^ (227) = 458.784, p < 0.001, CFI = 0.926, RMSEA = 0.071, SRMR = 0. 042); 1-factor solution (χ^2^ (299) = 731.614, p < 0.001, CFI = 0.878, RMSEA = 0.084, SRMR = 0.049)). In addition, we compared the chi-square model fit between the model based on the seven components and the models reported in literature using a chi-square difference test. Our model showed significantly better fit compared to all others (*p* < 0.001). Since the SPAI-C differentiates between physiological symptoms before (item 25) and in a social situation (item 26), we also checked whether an 8-factor solution with differentiated physiological symptoms adds value. Although fit indices were as good as for the solution based on the seven components, the goldbricker procedure [68] showed that item 25 and item 26 were redundant. The goldbricker procedure checks whether two components show similar correlations with other variables [47, 68]. Based on a minimum correlation of *r* = 0.80 and a threshold of 0.25, it was indicated that item 25 and item 26 correlate higher than *r* = 0.80 and have less than 25% different correlations, suggesting a huge thematic overlap. Therefore, we decided to include item 26 (physiological symptoms during a social situation*)* as Clark and Wells’ (1995) model refers to physiological symptoms during a social situation. Concerning the resulting seven items, the goldbricker procedure did not indicate any redundancy. Descriptive statistics of all items and an overview of items assigned to components (symptoms and cognitive variables) derived from theory are reported in the supplements.

**Table 2:** Representative items of the 7 factor-solution

Item^a^	7-factor-solution
S3: Scared while doing something and being observed	Observational fear
S5: Scared when answering questions in class or at group meetings	Performance fear
S7: Scared to meet new kids	Interactional fear
S21: Before going to a party, I think about what might go wrong	Negative expectation
S24: When I am with other people, I think “scary” thoughts	Dysfunctional cognition
S19: I avoid social situations	Avoidance behavior
S26: When I am in a social situation, I feel (somatic symptoms)	Physiological symptoms

^a^S3–S26: Items of the SPAI-C (Social Phobia and Anxiety Inventory for Children) that had the highest factor loading in the confirmatory factor analysis and were therefore included as representative items in the network analysis

### Network Analysis

In order to assess our first assumption of a positively connected symptom network, an undirected symptom network using a Gaussian graphical model (GGM) was estimated [69], based on the seven items of the SPAI-C. The sample size is sufficiently large enough to include seven nodes as it is within the recommendation of 3 subjects per calculated parameter (with n = 204, a maximum of 11 symptoms could be included) [36]. A least absolute shrinkage and selection operator (Lasso) regularization was applied, which removes weak links from the network and thus improves the power and interpretability of the network since fewer parameters need to be estimated. The used graph package automatically calculates 100 different networks to check which network performs best [39]. To obtain the network model with the best data fit, the extended Bayesian information criterion (EBIC) was applied, where the hyperparameter γ was set to 0.5 according to the recommendations in the literature [38, 70]. Since some of the SPAI-C items are ordinally scaled, the cor_auto function was used to calculate polychoric or polyserial correlations for ordinal variables [39]. Visualization was based on qgraph [65] package using the Fruchterman–Rheingold algorithm by default [39]. To assess our second assumption that cognitive variables take a central role within the SAD network and that dysfunctional cognitions act as a hub, we investigated connections between nodes (edge-weights) as well as at strength of nodes. Each node of the network represents one of the seven included components (symptoms or cognitive variables). Strength is defined as the sum of all absolute edge-weights of a single node and thus a measure for how well a node directly connects to other nodes. Additionally, we calculated node predictability, which indicates how much variance of a single node is explained by all other nodes [41, 42]. While centrality is a relative measure that indicates how important one component is compared to the other components of the network, predictability can absolutely indicate how adequately a component can be predicted by all other components [41, 42]. As the visualization of networks can be misleading regarding the edge-weights and the centrality of individual nodes, it is important to assess the accuracy of the network [39]. This was done by calculating bootstrapped difference tests between edge-weights and the three different centrality measures using a non-parametric bootstrap procedure with 1000 bootstrap samples. As centrality measures should only be interpreted if they can be considered stable, the network’s stability was assessed using a case-dropping bootstrap procedure with 1000 bootstrap samples and subsequently calculating the correlation stability coefficient (cs-coefficient) [39]. In this procedure, multiple networks with a decreasing number of included cases are estimated, and their correlation with the original network is calculated. The cs-coefficient indicates the proportion of people that can be dropped from the sample while remaining a set correlation (here 0.7) with the original network with a 95% probability. It has been recommended that ideally, the cs-coefficient should reach a value of > 0.5. However, centrality measures can still be considered stable and thus interpretable if they are > 0.25 [39].

## Results

**Fig. 1:**
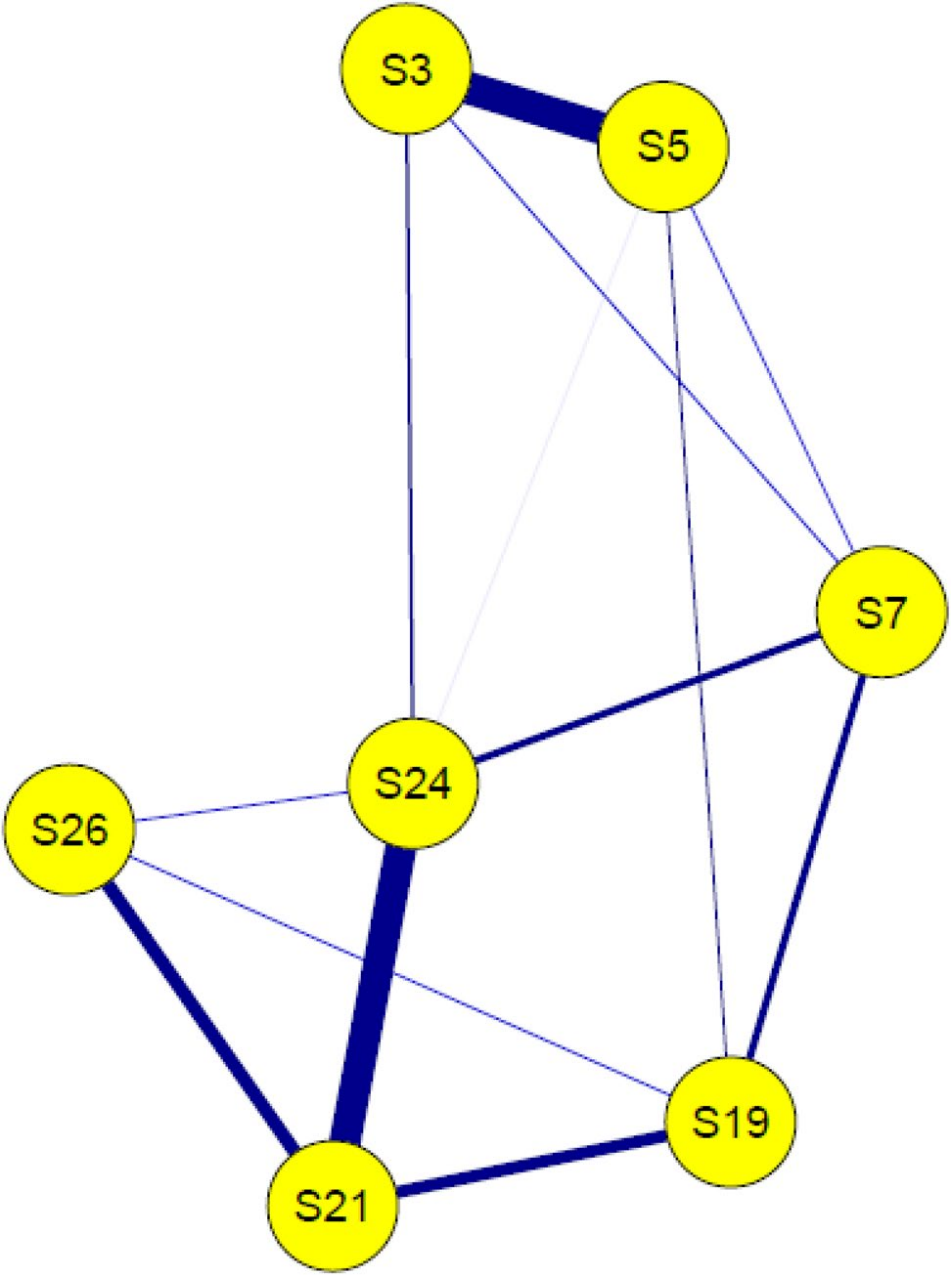
Network of included symptoms and cognitive variables. S3–S26: Items of the SPAI-C that had been included in the network analysis. SPAI-C = Social Phobia and Anxiety Inventory for Children; Nodes: S3 = Observational fear, S5 = Performance fear, S7 = Interactional fear, S19 = Avoidance behavior, S21 = Negative expectation, S24 = Dysfunctional cognition, S26 = Physiological symptoms

**Fig. 2:**
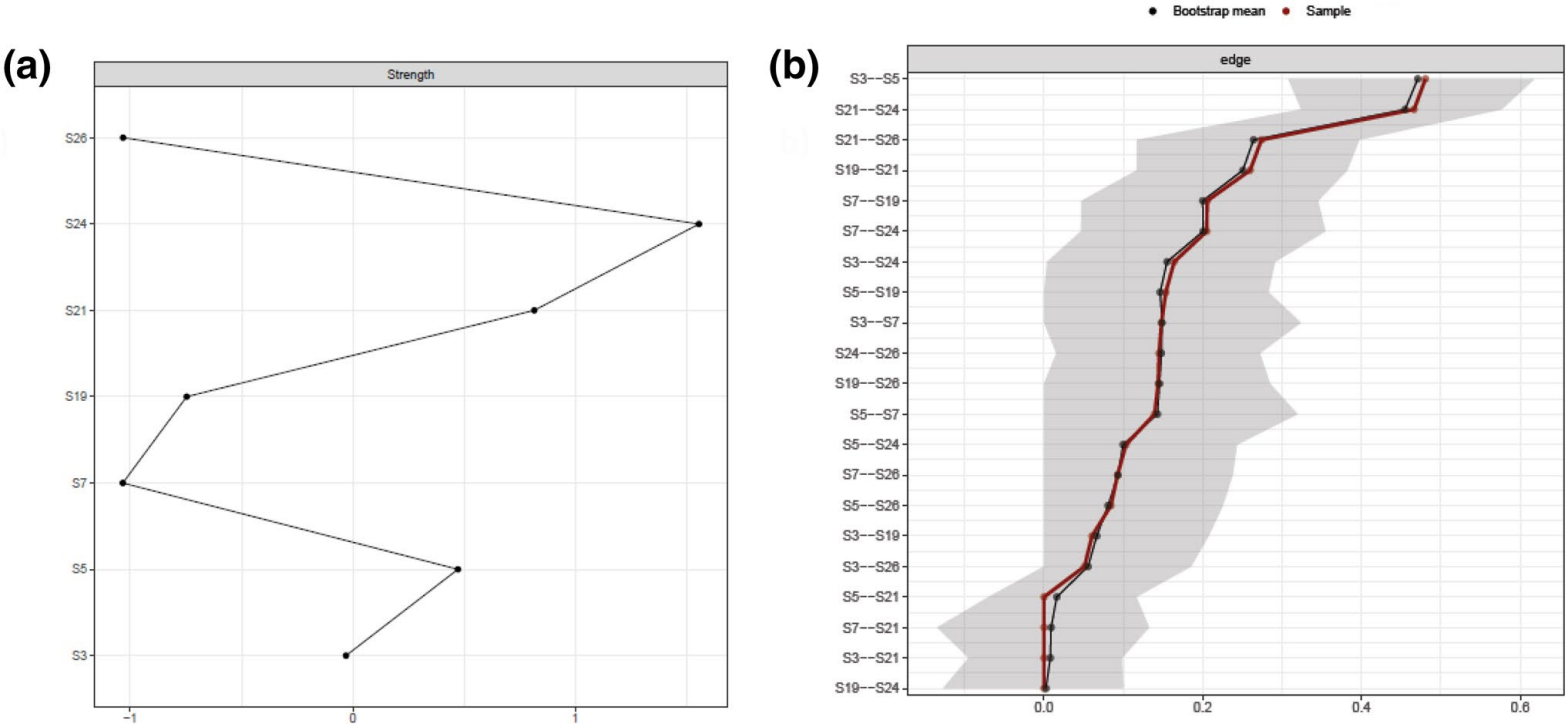
Strength per node and confidence intervals of edges. **a** Strength of nodes is shown here as a z-standardized value; **b** Confidence intervals for every edge are represented by grey area around lines. Red line represents edge weights from sample mean and black line from bootstrap mean; S3–S26: Items of the SPAI-C that had been included in the network analysis; SPAI-C = Social Phobia and Anxiety Inventory for Children

The SAD network is displayed in Fig. 1. Edges (links between nodes) represent partial correlations. In line with our first assumption, all edges of the network are positive. The confidence intervals (Fig. 2b) and difference tests (Supplementary Material) regarding the edge weights confirm the strength of the association between nodes S21 (negative expectations) and S24 (dysfunctional cognitions in social situations) and S3 (observational anxiety) and S5 (performance anxiety) shown in Fig. 1, as their associations are significantly stronger than the associations between almost all other nodes (Fig. 2). The only other edge showing a significantly stronger relationship than at least one other edge was that between nodes S21 (negative expectations) and S19 (avoidance behavior) (Supplementary Material).

Stability analysis showed that strength (cs-coefficient = 0.256) was stable enough to be interpreted, albeit with caution, as the cs-coefficient barely exceeds the minimum threshold of 0.25. Strength for the individual nodes is shown in Fig. 2a. In line with our second assumption, especially S24 (dysfunctional cognitions) proves to play a central role in the symptom network, as it has the highest strength of all nodes. Additionally, S21 (negative expectations) and S5 (performance fears) seem to have a descriptively higher strength concerning the other nodes of the network, indicating that they also play an important role within the symptom network.

The significance tests regarding the strength of each node (supplement material) indicate that only S24 (dysfunctional cognitions) has a higher strength than S19 (avoidance) and S26 (physiological symptoms).

The analysis regarding the predictability of the nodes showed that, on average, 57% of the variance of individual components can be explained by the components of the network connected with it. The variance resolution per node is displayed in the supplements. Remarkably, dysfunctional cognitions (R^2^ = 71%) and negative expectations (R^2^ = 69%) showed the highest amount of explained variance.

## Discussion

In the study at hand, we aimed to model a symptom network of SAD in children and adolescents and to investigate the role of cognitive variables within this network. We were able to provide additional evidence that SAD can be conceptualized as a positively interconnected network of symptoms in children and adolescents, as shown before by Miers et al. (2020) [52], based on a community sample of children and adolescents. In this respect, SAD symptoms and cognitive variables in children do not seem to occur in isolation, but could, according to the assumption of network theory [35] and according to the assumption of Clark and Wells’ (1995) model, influence and activate each other and constitute a stable symptom network. Although it is important to emphasize that the present network is based on cross-sectional data and thus no causal directionality can be inferred between the individual components, the parameters such as predictability and strength may be indicative of the importance of individual components of the network. Consistent with previous research showing that internalizing disorders in particular exhibit high predictability and thus high variance resolution by variables within the network (on average: 42%) [42], the SAD network at hand also shows a high average predictability (average variance resolution: 57%). This means that the occurrence of symptoms and cognitive variables is strongly predicted by other symptoms and cognitive variables of the network, which implies the assumption that neighboring components can be used in therapy to influence other components [42]. In contrast, other disorders with low predictability (e.g., psychosis: 28%) are more likely to be influenced by variables outside the symptom network (e.g., genes) [42].

### Importance of Cognitive Variables

We were also able to show that cognitive variables have a high impact within the SAD network in children and adolescents, which is indicated by the finding that the two cognitive variables showed the highest predictability and a high strength. This is consistent with findings of the study by Miers et al. (2020) [52], who were able to show that dysfunctional cognitions play a central role in the symptom network of SAD. In contrast to Miers et al. (2020) [52], we differentiated between different cognitive variables and found that especially dysfunctional cognitions during a social situation were associated with most other investigated symptoms. In line with the study by Miers et al. (2020) [52], our symptom network also shows no direct connection between dysfunctional cognitions and avoidance behavior, which was the only symptom to which the variable dysfunctional cognitions was not connected. In contrast, negative expectations about social situations were more specifically and strongly associated with fewer components, particularly dysfunctional cognitions, physiological symptoms, and avoidance behavior. This indicates a strong mutual connection between different cognitive processes and an association between cognitions occurring *before* a social situation and an actual avoidance of a social situation. Our results suggest that important variables of cognitive models of SAD, which have been formulated and already empirically tested for adulthood, also play a central role within the symptom network in children and adolescents. Similar to previous empirical studies [14, 17], this suggests that these models are at least partially valid for children and adolescents as well. Thus, SAD seems to be strongly shaped by cognitive mechanisms already in childhood and adolescence. In contrast to previous findings [14, 17, 24], our network further provides an opportunity to take a more nuanced look at the interplay between SAD symptoms and selected cognitive variables, such as negative expectations of social situations and dysfunctional cognitions in social situations. Unfortunately, we could also not include other variables of cognitive models such as post-event processing or self-focused attention in the network analysis because the analysis was based on an existing data set, and no data were available in this regard. Regarding the included cognitive variables, we found one of the strongest connections of our network between negative expectations about and dysfunctional cognitions in a social situation (see Fig. 2). Since these two cognitive variables both loaded on different factors in our CFA and were recognized as sufficiently different by the goldbricker procedure, it can be assumed that they are two distinct variables with different connections to SAD symptoms, as postulated by the SAD models [12]. As initial studies using regression analyses have already shown that these two cognitive variables are strongly related [23, 24], we confirmed this connection using the network approach.

### Dysfunctional Cognitions

Most strikingly, dysfunctional cognitions seem to be central to the network as they are linked to all other components of the network (except avoidance behavior). It is important to mention that although this variable has numerous connections to other components, which also results in the high strength value and high predictability of dysfunctional cognitions, only the connection to negative expectation is particularly strong. This may suggest that dysfunctional cognitions have more of a nonspecific and global connection on symptoms of SAD. Overall, these findings are consistent with the assumptions of Clark and Wells’ (1995) cognitive model, in which expectations of social situations activate dysfunctional cognitions and these, in turn, act as a hub affecting several other components (e.g., physical symptoms). In line with Leigh and Clarks’ (2016) [31] idea of implementing concrete variables of cognitive models in therapy to improve their effectiveness in children and adolescents, addressing dysfunctional cognitions in the treatment of SAD seems essential. The high centrality of dysfunctional cognitions in our study is also consistent with findings from studies in larger community samples showing that dysfunctional cognitions were one of the strongest predictors of social anxiety [23], however inconsistent with the mixed findings regarding the frequency of dysfunctional cognitions in children and adolescents with SAD [17]. The conceptualization of psychopathology in terms of the network approach could contribute to explain these findings. In this context, it is not necessarily the frequency but the influence of a symptom on other symptoms within the network and thus the connectivity between symptoms that is of importance [35]. Thus, one could hypothesize that dysfunctional cognitions are not necessarily more frequent in children and adolescents with SAD compared to healthy individuals, although they may be stronger associated with other symptoms. Consistently, Heeren et al. [45] found no qualitative difference between the network of SAD symptoms in healthy and affected adults. However, the connections between nodes were stronger in individuals with SAD. In this regard, future research should focus on a comparison of a symptom network between individuals with and without SAD.

### Negative Expectations

In contrast to the global connection of dysfunctional cognitions to components of the network, negative expectations are relatively specific but strongly linked to dysfunctional cognitions, avoidance behavior, and physiological symptoms. The link between negative expectations of social situations and avoidance behaviors, despite its lesser strength within the network compared to dysfunctional cognitions, may indicate a prominent role in the maintenance of SAD. Since negative expectations but not dysfunctional cognitions in the social situation show an association with avoidance behavior, negative thoughts prior to a social situation seem to be relevant for the behavioral symptomatology of SAD. For example, Clark and Wells’ (1995) model actually predicts that negative expectations of social situations are associated with avoidance of these situations [16] and thus contribute to the disorder’s maintenance since negative assumptions cannot be disconfirmed. Given that we cannot infer causal direction from our network, an influence from avoidance to negative expectation could also be possible. Here, a long-term avoidance of social situations could lead to the formation of negative expectations about these, as few (positive) social experiences are obtained. To our knowledge, no research has examined this relationship from which causal assumptions could be derived. In this context, longitudinal network analyses including components of SAD, such as avoidance and cognitive variables, would be essential to examine the development of the symptom network over time. Regardless of the causal direction between the two nodes, our network indicates that there is a close relationship between negative expectations and avoidance behaviors, which might be important to emphasize in the therapy of SAD because avoidance behavior is considered as important for the maintenance of anxiety disorders [71]. In particular, the use of in vivo behavioral experiments that aim at challenging negative expectations by attending real social situations seems promising in this context. This technique, already used by some CBT programs for SAD [14, 31], involves formulating specific predictions for social situations (e.g., “if I say something, the other children will laugh”) and recording how likely the event is to occur (e.g., 90%). In systematic behavioral experiments (e.g., saying something in the presence of other children), it is checked whether this prediction was correct and whether the probability of the event was realistic. While CBT has already been proved to be effective for children and adolescents with SAD [32], future research should focus on individual components, such as behavioral experiments, in order to find out which components are most effective. Although we cannot assume causal direction from our results, these findings may provide preliminary evidence that behavioral experiments promise to reduce avoidance behavior by targeting negative expectations. Consistently, the high predictability (R^2^ = 0.69) of negative expectation in the current network suggests that it could be a cognitive variable well suited to influence neighboring symptoms.

### Social Fears

At the level of concrete social fear, all three fears (performance, interaction, observation) were mutually connected, which indicates that they might influence each other. This is consistent with findings that individual social fears tend to occur in combination [11]. Further, performance and observational fears formed a strong link, whereas interactional fears were less strongly connected to the other two fears. While there are findings that interactional and performance fears lie at the heart of different subtypes of SAD [11], the role of observational fears in this context is less clear. For example, in the study of Knappe et al. (2009) [11], this fear was not explicitly compared to the other two fears regarding clinical features. Our study might suggest that observational fear is closely related to performance fear and thus might share similar clinical features. Since the importance of individual social fears for SAD depends on the developmental stage [11, 72], it would be important for future research to examine networks of SAD in different age groups. In our sample, we had a broad age range of 8–18 years but a limited amount of individuals, so that the influence of concrete fears in our network could not be investigated.

### Physiological Symptoms

Regarding the role of physiological symptoms within the SAD network, we neither found significantly stronger links to other symptoms nor a major influence on the network in general. While this may suggest that physiological symptoms play a comparatively minor role in SAD in children and adolescents, experimental research also indicates that children with SAD do not necessarily show a stronger physiological reactivity towards social stress [73]. However, it is important to note that the physiological symptoms included in our network analysis are based on subjective data. Research suggests that socially anxious children systematically overestimate their physiological symptoms [74]. Accordingly, future networks of SAD should differentially incorporate both an objective physiological measure and perceptions of physiological symptoms to account for the differential effects of these components.

### Strengths and Limitations

Our study has several strengths and limitations. First, the model of Clark and Wells (1995) [12] and previous research on cognitive variables of SAD in children and adolescents indicate that, in addition to the variables examined here, other mechanisms are central. Here, self-focused attention, post-event processing, and safety behavior are important variables that are known to be involved in SAD symptomatology and which we were not able to include, because they were not assessed. Of the centrality measures for the SAD network, only strength was stable and thus interpretable, although even this did not reach the ideal threshold of 0.5. Second, it is important to emphasize that there is an ongoing controversy in the literature regarding the utility of network analysis and how its results can be interpreted, with some researchers arguing that associations between symptoms do not represent meaningful associations or are due to artifacts [75], and others arguing that networks are highly replicable and thus found associations cannot be mere artifacts [76]. One criticism, for example, is that in networks based on cross-sectional data, it is not possible to disentangle clearly whether associations between symptoms actually represent a meaningful association or are due to artifacts of, for example, shared item content or method variance [75]. Network analysis represents a new approach that is constantly evolving, and we have taken advantage of current methodological possibilities to counteract possible error artifacts (for example, empirical item selection and goldbricker procedure to eliminate overlapping items). Nevertheless, it is important to emphasize that the present network does not allow for conclusions to be drawn about directional connections and possible developments of symptoms over time and should rather be considered as a source for forming hypotheses about connections of symptoms and variables [77]. Third, we included children of a wide age range of 8–18 years in the current study. Therefore, the network at hand does not provide any information regarding the variation of symptoms and cognitive variables across developmental stages. It is important to emphasize that far-reaching cognitive developments take place during late childhood and adolescence, which appear to be due to an interaction between changes in neural circuitry and changes in the social environment [78]. Future studies should particularly focus on developmental changes and consider different age groups of children and adolescents when examining cognitive variables. Fourth, we used a mixed sample of individuals with low and high social anxiety and did not analyze the network in a homogenous group of SAD patients, which might limit generalizability to clinical populations. While studies in adults suggest that the network structure does not differ between patients and healthy individuals, there is no empirical evidence for this in children and adolescents. However, since anxiety occurs normatively in childhood and adolescence and only becomes clinically relevant once it reaches a certain level or duration [3], it seems likely that there are no differences between patients and healthy individuals here either. In addition, the numerous high-socially anxious individuals, the relatively high variability (*SD* = 13.53), high amount of explained variance of symptoms, as well as relatively high mean SPAI-C sum score (*M* = 20.00) of our sample, which on average even exceeds the clinical cut-off of the SPAI-C, suggest a sufficient variance regarding the SAD components of our sample. Also, the circumstance that some of the children additionally score higher on an SM screening is probably not problematic due to findings that children with SM do not differ from children with SAD with respect to fear-related cognitions [54] and due to high comorbidity rates between both disorders [58]. Nevertheless, future studies should compare network structures of SAD between a clinical and healthy group in children and adolescents to prove the assumption of dimensionality of social anxiety also for network analysis.

The study’s strengths are, first, that we were able to show that cognitive variables from Clark and Wells’ (1995) cognitive model of SAD also represent central elements of SAD in children and adolescents in a sample with a substantial amount of highly socially anxious individuals. This suggests that this model of SAD, created initially based on adult patients with SAD, is at least partially valid in children and adolescents. The advantages of network analysis compared to correlational studies are the quantification of the centrality of individual variables compared to other symptoms within the network and the identification of specific links between symptoms. Thus, we gained a fine-grained insight into the importance of cognitive variables and specific links between cognitive variables and other symptoms of the SAD network, which allowed us to derive possible therapeutic implications. Second, we conducted a combination of theory- and data-driven item selection in advance to address methodological concerns about unregulated networks. Here, the resulting factor solution proved superior to previous factor solutions, and there was no evidence of conceptually overlapping symptoms. This suggests that the calculated network includes central symptoms of SAD in childhood and adolescence and that centrality measures are not distorted symptoms, which are too similar.

## Conclusion

In conclusion, the study at hand provides evidence that SAD can be conceptualized as a positively interconnected network of symptoms in children and adolescents. The high amount of explained variance among symptoms could suggest that targeting central symptoms in the treatment of SAD might be associated with a significant impact on the other symptoms of the disorder. We further showed that the cognitive variables negative expectation and dysfunctional cognitions have a differential effect on the symptom network of SAD. While dysfunctional cognitions seem to have a global influence on many different symptoms of SAD, negative expectations are strongly and more specifically connected to few symptoms, including avoidance of social situations. The results could implicate that targeting cognitive variables differentially in therapy might have different effects on symptoms of SAD.

## Summary

In the present study, we investigated which role cognitive variables of Clark and Wells’ (1995) cognitive model of social anxiety disorder (SAD) play in children and adolescents and how they relate to other symptoms of SAD. For this, we used the innovative network approach, which views mental disorders as networks of mutually activating symptoms. The analysis allows to gain a fine-grained insight into the interrelationships of individual symptoms and variables and to quantify their importance within the network. The present study, which was based on a mixed sample of high- and low-socially anxious children and adolescents, provides the second symptom network that exists with children and adolescents in the context of SAD. In contrast to the first network study by Miers et al. (2020), we differentiated on the two cognitive variables of SAD negative expectations and dysfunctional cognitions. The fine-grained examination of cognitive variables in children and adolescents in the context of SAD is of central importance as specific targets of therapy can be derived from this. Although cognitive variables, in general, are already addressed in cognitive behavioral therapy, little is known about the relationship between specific cognitive variables components and symptoms of SAD in children and adolescents. We were able to show that SAD can also be presented in children and adolescents using a symptom network. While dysfunctional cognitions had the strongest influence within the network and were associated with almost all symptoms, negative expectations about social situations were more specifically associated with fewer symptoms, including avoidance behavior. On the one hand, our results might suggest that dysfunctional cognitions act as a central hub during a social situation and activate other SAD symptoms, as postulated in cognitive models of SAD. On the other hand, negative expectations of social situations might play a special role in the maintenance of SAD because of the link to avoidance of social situations. Overall, the study suggests that a differentiated consideration of specific cognitive variables appears important in therapy for children and adolescents.

## Supplementary Information

Below is the link to the electronic supplementary material.

Supplementary file1 (DOCX 54 kb)
