# Supplementary material for: Cognitive Variables in Social Anxiety Disorder in Children and Adolescents: A Network Analysis
Source: Child Psychiatry Hum Dev. 2021 Oct 27;54(3):625–38. doi: 10.1007/s10578-021-01273-9 (PMC10150579; doi:10.1007/s10578-021-01273-9)
Supplement: Supplementary file 1 — Supplementary file1 (DOCX 54 kb) [file 10578_2021_1273_MOESM1_ESM.docx]

| 7-factor solution |  |
| --- | --- |
| Observational fear | Item 2: Scared when becoming the center of attention |
|  | **Item 3**: Scared when doing sth and others are watching |
| Performance fear | Item 4: Scared when speaking or reading in front of a group |
|  | **Item 5**: Scared when answering questions in class or at group meetings |
|  | Item 8: Too scared to ask questions in class |
|  | Item 16: Scared when speaking in front of class |
|  | Item 17: Scared when in a school play, choir, music or dance recital |
| Interactional fear | **Item 7**: Scared to meet new kids |
|  | Item 9: Scared in the school cafeteria |
|  | Item 14: Scared when I talk to someone |
|  | Item 15: Scared to talk to someone for longer than a minute |
| Avoidance behavior | Item 6: Scared at parties, dances, school… and go home early |
|  | **Item 19**: I avoid social situations |
|  | Item 20: I leave social situations |
| Negative expectations | **Item 21**: Before going to a party, I think about what might go wrong |
| Dysfunctional cognitions | **Item 24**: When I am with other people, I think “scary” thoughts |
| Physiological symptoms | **Item 26**: When I am in a social situation, I feel (somatic symptoms) |
|  | Item 25: Before going someplace, I feel (somatic symptoms) |
|  |  |

Table A: Identified symptom categories and assigned items

| Items | 7-factor solution | 8-factor solution |
| --- | --- | --- |
| Item: 2, 3 | Observational fear | Observational fear |
| Item: 4, 5, 8, 16, 17 | Performance fear | Performance fear |
| Item 7, 9, 14, 15 | Interactional fear | Interactional fear |
| Item: 6, 19, 20 | Avoidance behavior | Avoidance behavior |
| Item: 21 | Negative expectations | Negative expectations |
| Item: 24 | Dysfunctional cognitions | Dysfunctional cognitions |
| Item: 26 | Physiological symptoms | Item: 25 Anticipatory physiological symptoms |
|  |  | Situational physiological symptoms |

Table B: Items of both factor solutions

Table C: Descriptive statistics of our sample for all items of SPAI-C

|  | | | | | |
| --- | --- | --- | --- | --- | --- |
|  | N | Minimum | Maximum | M | SD |
| S1 | 204 | .00 | 2.00 | .80 | .78 |
| S2 | 204 | .00 | 2.00 | .96 | .74 |
| S3 | 204 | .00 | 2.00 | .89 | .84 |
| S4 | 204 | .00 | 2.00 | 1.11 | .80 |
| S5 | 204 | .00 | 2.00 | .83 | .86 |
| S6 | 204 | .00 | 2.00 | .37 | .64 |
| S7 | 204 | .00 | 2.00 | .77 | .77 |
| S8 | 204 | .00 | 2.00 | .67 | .82 |
| S9 | 204 | .00 | 2.00 | .64 | .56 |
| S10 | 204 | .00 | 2.00 | .95 | .61 |
| S11 | 204 | .00 | 2.00 | .84 | .64 |
| S12 | 204 | .00 | 2.00 | .95 | .60 |
| S13 | 204 | .00 | 2.00 | .90 | .65 |
| S14 | 204 | .00 | 2.00 | .84 | .63 |
| S15 | 204 | .00 | 2.00 | .69 | .60 |
| S16 | 204 | .00 | 2.00 | .86 | .66 |
| S17 | 204 | .00 | 2.00 | .81 | .73 |
| S18 | 204 | .00 | 2.00 | 1.11 | .72 |
| S19 | 204 | .00 | 2.00 | .68 | .61 |
| S20 | 204 | .00 | 2.00 | .64 | .58 |
| S21 | 204 | .00 | 2.00 | .63 | .62 |
| S22 | 204 | .00 | 2.00 | .54 | .70 |
| S23 | 204 | .00 | 2.00 | .85 | .81 |
| S24 | 204 | .00 | 2.00 | .68 | .62 |
| S25 | 204 | .00 | 2.00 | .48 | .52 |
| S26 | 204 | .00 | 2.00 | .39 | .49 |

Table D: Explained Variance of items included in the network analysis

Variable R²

S3: .49

S5: .52

S7: .47

S19: .57

S21: .69

S24: .71

S26: .60

Note: S3 – S26: Items of the SPAI-C that had the highest factor loading in the confirmatory factor analysis and were therefore included as representative items in the network analysis; SPAI-C = Social Phobia and Anxiety Inventory for Children

Figure A: Comparison of edge weights

Note: Black boxes indicate a significant difference between the compared edges; S3 – S25_26: Items of the SPAI-C that had been included in the network analysis; SPAI-C = Social Phobia and Anxiety Inventory for Children; Strength is shown here as a z-standardized value;

Figure B: Comparison of strength between nodes

Note: Black boxes indicate a significant difference between the strength of the compared nodes; S3 – S25_26: Items of the SPAI-C that had been included in the network analysis; SPAI-C = Social Phobia and Anxiety Inventory for Children; Strength is shown here as a z-standardized value;
